# Reliable assessment of pollen quality in *Chrysanthemum × morifolium*: comparative evaluation of viability tests and germination media

**DOI:** 10.1186/s12870-026-09032-5

**Published:** 2026-06-08

**Authors:** Tuğba Kılıç

**Affiliations:** Horticulture Department, Faculty of Agriculture, Yozgat Bozok University, Yozgat, Türkiye

**Keywords:** Chrysanthemum, TTC, PEG, Pollen germination, Pollen viability, Heat-treated pollen, SDG12

## Abstract

**Background:**

Reliable evaluation of pollen quality is essential for successful hybridization and breeding programs in *Chrysanthemum × morifolium*. However, commonly used staining-based pollen viability tests do not always reflect functional pollen performance, and the relationship between pollen viability estimates and actual germination capacity remains insufficiently clarified in chrysanthemum.

**Results:**

In this study, the reliability of pollen viability and germination tests was evaluated using three chrysanthemum cultivars (Chic, Swan, and Barolo). Five pollen viability tests [iodine-potassium iodide (IKI), 2,3,5-triphenyl tetrazolium chloride (TTC), lactophenol blue (LCB), safranin (SFN), and acetocarmine (AST)] and eight in vitro germination assays consisting of four liquid and four semi solid media were compared using hanging-drop and Petri dish agar methods. In total, thirteen tests were evaluated under both fresh and heat-treated (80 °C for 48 h) pollen conditions, with 1.000 pollen grains analyzed per cultivar. Staining-based tests such as SFN, IKI, LCB, and AST produced high apparent viability values (76–98%), whereas the TTC test yielded markedly lower values (4–8%). Despite these high viability estimates, pollen germination percentages remained generally low across all media (0.1–7.3%). The highest germination performance was observed in polyethylene glycol (PEG)-based liquid media, particularly S-4 and S-1. PCA revealed an association between TTC viability results and germination success.

**Conclusions:**

The results demonstrate a clear discrepancy between staining-based viability estimates and actual pollen germination capacity in chrysanthemum. The TTC test appeared to be the viability assay most closely associated with functional pollen performance under the tested conditions. TTC may provide a more functionally relevant estimate of pollen viability than staining methods such as IKI. The combined use of TTC-based viability testing and PEG-supported germination assays may therefore represent a promising approach for selecting highly functional pollen in chrysanthemum breeding programs.

**Supplementary Information:**

The online version contains supplementary material available at 10.1186/s12870-026-09032-5.

## Introduction

*Chrysanthemum × morifolium* Ramat., formerly classified as *Dendranthema grandiflorum* (Ramat.) Kitam., is a perennial flowering species belonging to the family Asteraceae and is widely used in the ornamental plant industry as well as in medicinal and cosmetic applications. Within the global ornamental plant sector, chrysanthemum ranks among the four most preferred cut flower species and contributes substantially to the international floriculture market, accounting for more than 30% of global cut flower production [1].

The broad utilization and high commercial value of chrysanthemum continue to drive breeders and researchers to develop new cultivars each year. Conventional breeding approaches, particularly hybridization and mutation breeding, remain the most widely applied strategies for cultivar development [2]. However, mutation breeding approaches—especially those involving physical mutagens—require specialized facilities and strict regulatory compliance, while chemical mutagens pose environmental and occupational health risks [3], limiting their broader application. Consequently, classical hybridization remains the dominant breeding strategy in chrysanthemum, aiming to generate genetic variation through controlled crossing of individuals with contrasting traits [2, 4]. Although advanced biotechnological tools such as genetic transformation and protoplast fusion offer alternative approaches for cultivar development, their high costs and technical complexity limit their widespread use [5, 6].

Hybridization is considered a simple and efficient breeding approach; however, the fertility of hybrid combinations represents one of the most critical limiting factors in this process [2]. Chrysanthemum is characterized by a highly heterozygous genome and a complex genetic background, which may limit classical breeding efforts. Long-term breeding activities— particularly intensive selection, the repeated use of a limited genetic pool, and breeding practices including mutation breeding— may have contributed to inbreeding depression and the accumulation of deleterious genetic load within breeding populations, potentially reducing overall fertility [7, 8]. Moreover, the sporophytic self-incompatibility (SI) system prevents self-fertilization and limits inbreeding; however, the extensive sharing of S-alleles among modern cultivars also results in fertilization failure even between genetically distinct individuals. This phenomenon markedly restricts seed formation in chrysanthemum [9–12]. In addition, the inability to produce viable pollen or the failure of pollen to germinate on the stigma and style constitutes another major barrier to successful seed set following hybridization [8].

The development of male and female reproductive organs in chrysanthemum has been extensively investigated, and the functional interactions between these organs during fertilization are well described [13]. In many plant species, pollen viability estimated by staining-based tests does not necessarily correspond with actual pollen germination capacity, creating uncertainty in the evaluation of functional pollen performance. However, studies specifically addressing pollen viability in chrysanthemum remain limited [3, 8]. The lack of clearly established, reliable, and easily applicable methods for evaluating pollen viability and germination in potential male parents constitutes a major bottleneck limiting the higher seed yields in modern chrysanthemum cultivars. Within this framework, the present study aimed to evaluate the accuracy and reliability of commonly used test methods for determining pollen viability and in vitro germination capacity in three cultivars of *Chrysanthemum × morifolium*. In addition, the study sought to identify the most suitable and viability and germination assays for chrysanthemum breeding programs.

## Materials and methods

**Fig. 1:**
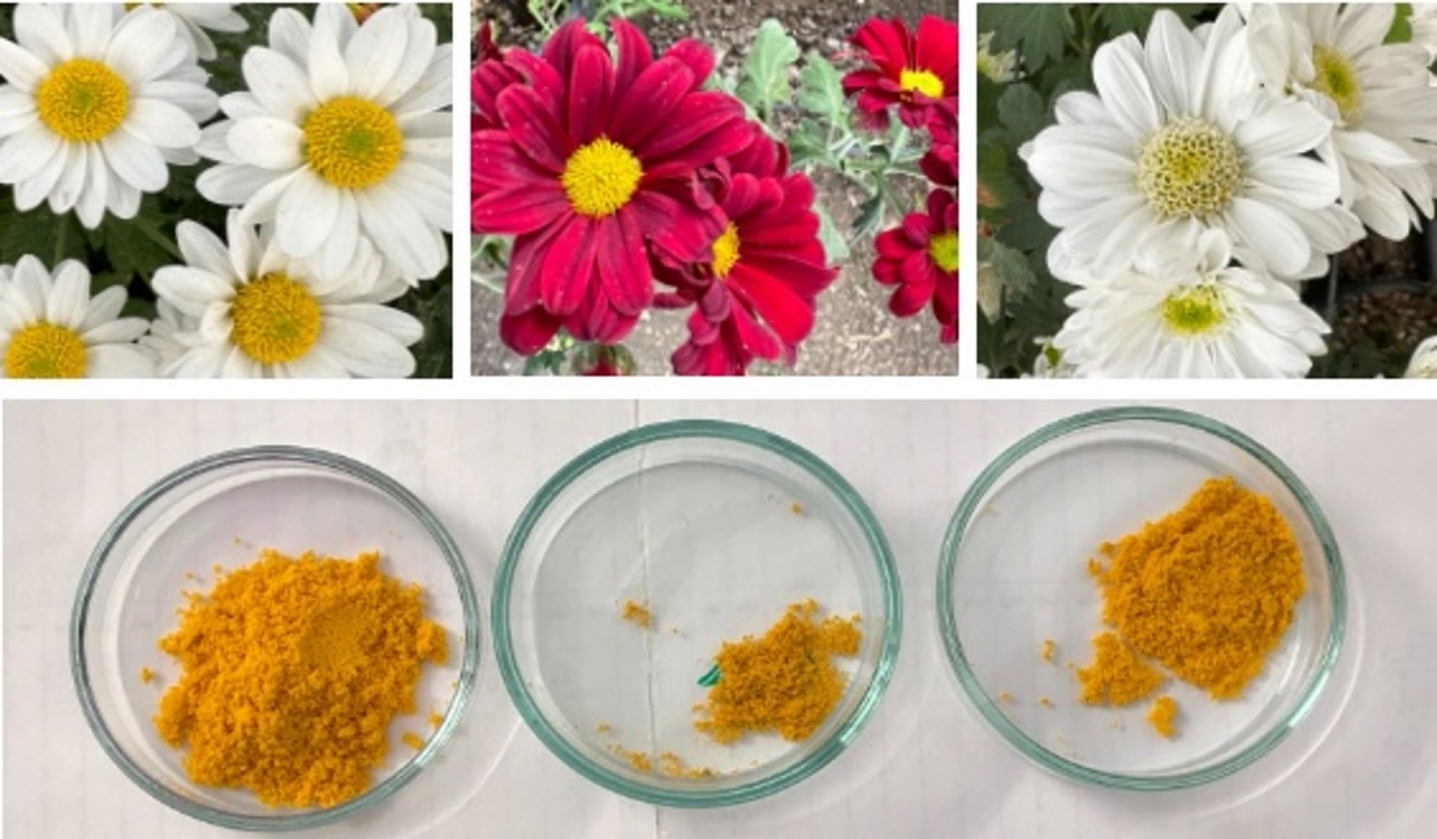
Flowering stage of chrysanthemum cultivars at the time of pollen collection (Chic, Barolo, Swan respectively) and pollen grains of chrysanthemum cultivars

The plant material consisted of pollen grains (Fig. 1) obtained from three cultivars (Barolo, Chic, and Swan) of *Chrysanthemum × morifolium* Ramat., grown between April and September in the cut flower breeding greenhouse of the Department of Horticulture, Faculty of Agriculture, Ankara University (39°57′40″N, 32°51′51″E). Seedlings of the parental chrysanthemum genotypes were transplanted under soil-based greenhouse conditions into raised beds measuring 1 m x 20 m, arranged in eight rows with 10 cm between rows and 12 cm between plants within rows.

During the growing period, the daytime greenhouse temperature was maintained at 25 ± 5 °C and relative humidity at 65–75%. Shading was provided using both thermal screens and shading compounds applied to the roof and side walls of the greenhouse to reduce excessive light intensity. Irrigation and fertilization were performed via an automated drip fertigation system for 20–40 min at 2-4-day intervals. The electrical conductivity (EC) of the nutrient solution was adjusted to 1.4 mS cm⁻¹ beginning one week after transplanting, increased to 1.8 mS cm⁻¹ when plants reached 35–40 cm in height, and further raised to 2.0 mS cm⁻¹ from the bud formation stage to flowering. The pH of the nutrient solution was maintained at 6.5 throughout the experimental period. The macro- and micronutrient composition of the nutrient solution is presented in Table 1.

**Table 1:** Ionic composition of the nutrient solution used in chrysanthemum cultivation (14], personal communications)

Elements	Concentration	Elements	Concentration
Macronutrients (mmol L⁻¹)		**Micronutrients (µmol L⁻¹)**	
NO₃⁻	5.0–8.0	Fe	40
NH₄⁺	0.1–0.2	Mn	2.0–3.0
H₂PO₄²⁻	0.8–1.2	Zn	2.0–3.0
SO₄²⁻	1.0–1.4	B	20
K⁺	3.0–4.0	Cu	1.0
Ca²⁺	3.0–4.0	MoMo	1.0
Mg²⁺	0.8–1.1	-	-

When plants reached approximately 30–35 cm in height, short-day conditions were induced by providing at least 13 h of darkness per day for approximately 7–8 weeks, and the short-day treatment was terminated at the onset of flower coloration. To ensure upright growth and prevent lodging, a four-layer support net was installed on the beds. At the coloration stage, the main flower buds were removed manually. Disease and pest management were carried out using an integrated plant protection approach, combining cultural practices, biotechnical methods (yellow sticky traps), and chemical control when necessary. Fungicides were applied against major fungal diseases such as rust, downy mildew, and Botrytis, while selective insecticides and acaricides were used against key pests including thrips, aphids, leaf miners, and spider mites [15].

Flower stems were harvested at the stage when the flowers were fully open (Fig. 1). The harvested stems were immediately placed in buckets containing tap water and transported to the laboratory within 30 min. Upon arrival, the flowers were inverted over glass Petri dishes, and pollen grains were released into the dishes by gently tapping the flower heads. The collected pollen was used to determine pollen viability and in vitro germination capacity. Both fresh and heat-treated pollen (80 °C for 48 h) were used in the viability and germination assays. For the heat treatment, pollen samples were placed in glass Petri dishes and maintained in a temperature-controlled incubator to ensure uniform heat exposure and to induce loss of pollen viability.

**Fig. 2:**
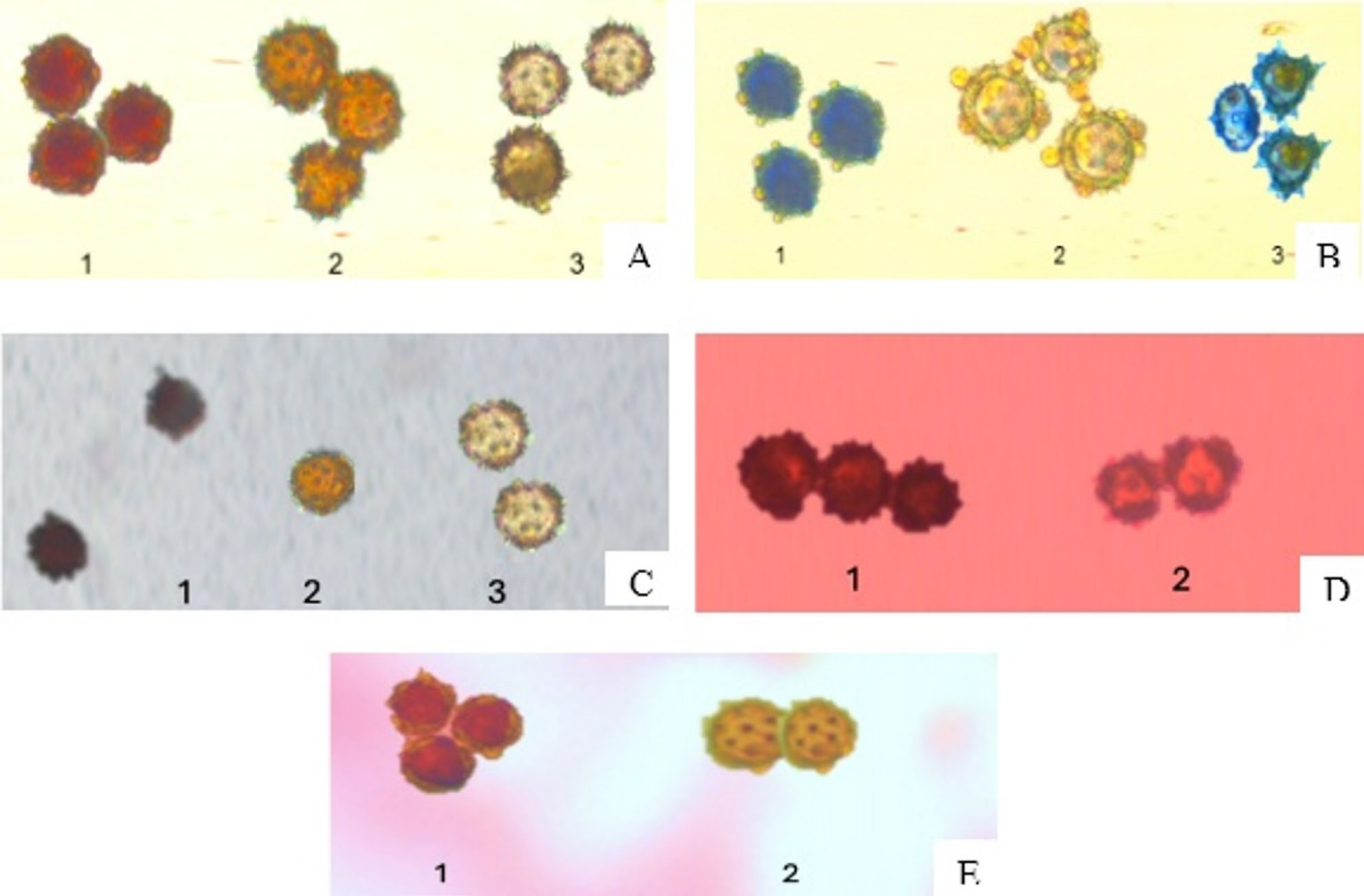
Pollen grains classified as absolutely viable (1), semi-viable (2), and non-viable (3) according to the TTC method (**A**). Pollen grains classified as viable (1), non-viable (2), and abnormal (3) according to the LCB method (**B**). Pollen grains classified as absolutely viable (1), semi-viable (2), and non-viable (3) according to the IKI method (**C**). Pollen grains classified as viable (1) and non-viable (2). according to the SFN method (**D**). Pollen grains classified as viable (1) and non-viable (2) according to the AST method (**E**)

Pollen viability was evaluated using IKI, TTC, LCB, SFN, and AST staining assays (Fig. 2). For each assay, pollen grains were spread onto microscope slides containing a drop of the appropriate staining solution and covered with a coverslip. In the IKI method, pollen grains that stained dark brown to black were classified as fully viable; those that stained light brown, orange, or red were considered semi-viable; and pollen grains that appeared yellow or colorless were categorized as non-viable. Microscopic evaluation was performed 4–5 min after staining [16]. In the TTC test, pollen grains exhibiting dark red or red coloration were classified as viable, while those that stained light red, reddish-brown, or pink were regarded as semi-viable, and yellow-stained or unstained grains were considered non-viable. The slides were incubated in darkness at 70% relative humidity for 12 h prior to evaluation [17]. In the LCB method, viable pollen grains appeared dark blue, whereas non-viable grains showed light blue, yellowish, or colorless staining. Slides were incubated in the dark at 70% relative humidity for 30 min before microscopic examination [18, 19].

In the SFN assay, pollen grains staining dark red were classified as viable, while pink-stained or unstained grains were classified as non-viable. The slides were maintained in darkness at 70% relative humidity for 1 h before evaluation [16, 20]. In the AST method, pollen grains staining dark red were considered viable, whereas light yellow-stained or unstained grains were classified as non-viable. Slides were incubated at 70% relative humidity for 3 h prior to microscopic evaluation [21].

In the calculation of percentage viability, semi-viable pollen grains were defined as those exhibiting partial staining and cytoplasmic integrity, representing an intermediate status between fully stained (viable) and unstained (non-viable) pollen. These grains were considered 50% viable and 50% non-viable. The total number of viable pollen grains was calculated by adding half of the semi-viable pollen to the number of fully viable pollen grains. Percentage viability was then determined by dividing the number of viable pollen grains by the total number of pollen grains counted [22, 23]. Morphologically abnormal pollen grains were also recorded as non-viable (Fig. 2).

Pollen germination was assessed using two in vitro methods: the Petri dish agar method and the hanging drop method, both performed under identical incubation conditions. Pollen cultures were incubated at 20 °C and 75% relative humidity, and germination percentages were monitored at regular intervals. Microscopic observations were carried out after 12 h of incubation, prior to excessive elongation of pollen tubes that could interfere with counting. Pollen grains were considered germinated when the pollen tube length reached at least 1.5 times the diameter of the pollen grain.

**Fig. 3:**
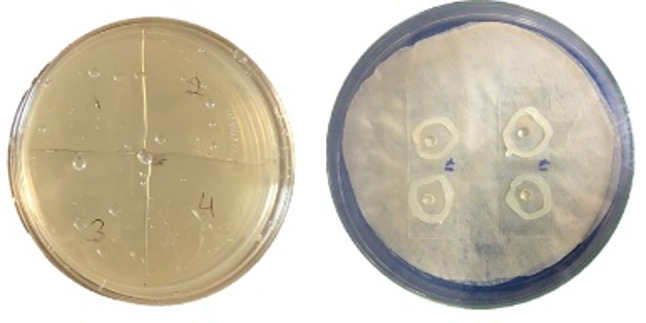
Petri dish agar method (left), hanging drop method (right)

In the Petri dish agar method (Fig. 3), solid media were prepared based on Brewbaker and Kwack (BK) medium, a modified ME3 medium, and an agar-based medium supplemented with boric acid, calcium nitrate, and sucrose (Table 2). The prepared media were poured into Petri dishes to a depth of approximately 2 mm and allowed to solidify. Each Petri dish was divided into four equal sectors, and pollen grains were evenly distributed onto each sector using a sable brush. To maintain internal humidity, the Petri dish lids were fitted with two layers of moistened filter paper.

**Table 2:** Composition and pH characteristics of the culture media applied in the Petri dish agar

Culture Media	Components	pH
Brewbaker and Kwack (K-1)	10% sucrose + 100 mg/L boric acid + 300 mg/L calcium nitrate + 200 mg/L magnesium sulfate + 100 mg/L potassium nitrate + 0.5% agar.	5.8
Modified solid ME_3_ (K-2)	370 mg/L magnesium sulfate + 950 mg/L potassium nitrate + 85 mg/L potassium phosphate + 880 mg/L calcium chloride + 412.5 mg/L ammonium nitrate + 175 mg/L potassium chloride + 7.45 mg/L disodium EDTA + 5.55 mg/L iron (II) sulfate + 50 mg/L boric acid + 16.50 mg/L manganese (II) sulfate + 10.50 mg/L zinc sulfate + 0.83 mg/L potassium iodide + 0.25 mg/L sodium molybdate + 0.025 mg/L copper(II) sulfate + 0.025 mg/L cobalt (II) chloride + 1.0 mg/L thiamine + 1.0 mg/L pyridoxine + 100 g/L PEG4000 + 0.5% agar.	5.8
Agar medium-1 (K-3)	0.5% agar + 20% sucrose + 10 ppm boric acid.	5.8
Agar medium-2 (K-4)	0.5% agar + 20% sucrose + 10 ppm boric acid + 300 mg/L calcium nitrate.	5.8

In the hanging drop method (Fig. 3), liquid media were prepared using modified ME3 medium supplemented with either PEG1500 or PEG4000, as well as media containing sucrose and PEG1500 (Table 3). A drop of the liquid medium was placed onto a coverslip using a micropipette, and pollen grains were applied with a sable brush. The coverslip was subsequently inverted onto a cavity slide sealed with a vaseline ring to allow the droplet to remain suspended. The slides were then placed in Petri dishes lined with moistened filter paper and sealed to maintain humidity [24].

All microscopic examinations were performed using a Leica DM1000 light microscope equipped with a digital imaging system and objective lenses at ×20, ×40, and ×100 magnifications.

**Table 3:** Composition and pH characteristics of the culture media applied in the hanging drop techniques

Culture Media	Components	pH
Modified liquid ME_3_-1 (S-1)	370 mg/L magnesium sulfate + 950 mg/L potassium nitrate + 85 mg/L potassium phosphate + 880 mg/L calcium chloride + 412.5 mg/L ammonium nitrate + 175 mg/L potassium chloride + 7.45 mg/L disodium EDTA + 5.55 mg/L iron (II) sulfate + 50 mg/L boric acid + 16.50 mg/L manganese (II) sulfate + 10.50 mg/L zinc sulfate + 0.83 mg/L potassium iodide + 0.25 mg/L sodium molybdate + 0.025 mg/L copper (II) sulfate +. 0.025 mg/L cobalt (II) chloride + 1.0 mg/L thiamine + 1.0 mg/L pyridoxine + 200 g/L PEG4000.	5.8
Modified liquid ME3-2 (S-2)	370 mg/L magnesium sulfate + 950 mg/L potassium nitrate + 85 mg/L potassium phosphate + 880 mg/L calcium chloride + 412.5 mg/L ammonium nitrate + 175 mg/L potassium chloride + 7.45 mg/L disodium EDTA + 5.55 mg/L iron (II) sulfate + 50 mg/L boric acid + 16.50 mg/L manganese (II) sulfate + 10.50 mg/L zinc sulfate + 0.83 mg/L potassium iodide + 0.25 mg/L sodium molybdate + 0.025 mg/L copper (II) sulfate + 0.025 mg/L cobalt (II) chloride + 1.0 mg/L thiamine + 1.0 mg/L pyridoxine + 300 g/L PEG1500.	5.8
Liquid culture medium (S-3)	20% sucrose + 10 ppm boric acid.	5.8
PEG culture medium (S-4)	200 g/L PEG1500 + 10 ppm boric acid.	5.8

A total of 13 different evaluation methods were applied for each cultivar. Due to practical limitations, it was not feasible to apply all available in vitro pollen quality assessment techniques. The selected methods were chosen based on their applicability under minimal laboratory conditions and their cost-effectiveness. Furthermore, optimization studies involving variations in medium components such as pH, sucrose concentration, or boric acid levels were not conducted. This limitation was mainly related to the necessity of using fresh pollen grains and the difficulty of performing viability and germination assessments simultaneously under microscopic observation. In viability tests, stained pollen grains needed to be evaluated within a short time period, as prolonged delays could result in structural damage or changes in staining characteristics, potentially affecting data accuracy. In germination tests, excessive incubation durations could lead to overly elongated pollen tubes, making microscopic evaluation difficult. Additionally, pollen viability is known to vary depending on the developmental stage of the flower and may fluctuate at different times of the day. Therefore, to ensure experimental reliability and consistency, standardized procedures based on commonly used methods for cut flower species were adopted.

To evaluate pollen viability and in vitro germination percentages, experiments were established using a Completely Randomized Design (CRD). For each cultivar, two coverslips were prepared, and two randomly selected microscopic fields were examined per coverslip. Approximately 250 pollen grains were counted in each field, resulting in approximately 1.000 pollen grains evaluated per cultivar. Multiple counts obtained from coverslips and microscopic fields were treated as technical replicates to improve counting precision and did not represent independent biological replicates. The experimental design was based on three independent biological replicates. Data were expressed as percentages, and angular (arcsine square root) transformation was applied prior to statistical analysis. Two-way analysis of variance (ANOVA) was conducted using IBM SPSS 24 software, and means were compared using Duncan’s multiple range test. In addition, Principal Component Analysis (PCA) was used as a complementary multivariate approach to explore relationships among variables. PCA was conducted using XLSTAT software, and the number of retained components was determined according to explained variance and scree plot evaluation. The results were visualized using biplots to facilitate interpretation of component structure and data distribution.

## Results

**Fig. 4:**
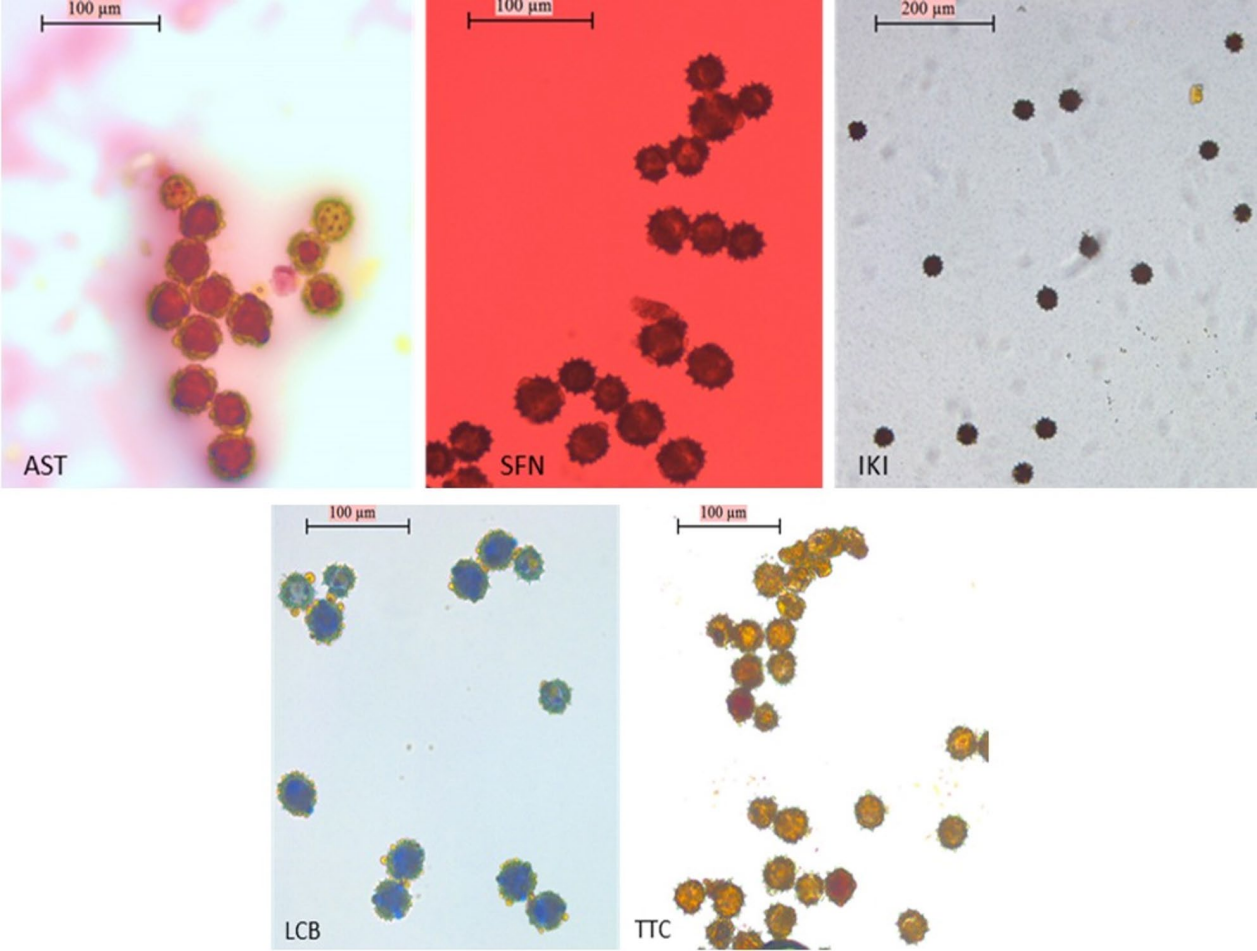
Chrysanthemum pollen grains stained with different staining methods

A two-way analysis of variance (ANOVA) showed that both cultivar × staining method and cultivar × germination medium interactions were significant (*p* < 0.05). The main effects of cultivar, staining methods, and germination media were also found to be significant (*p* < 0.05). The highest viability percentage (98%) was determined in the Barolo cultivar in the SFN test, and similarly high values were observed in the Chic cultivar, which belonged to the same statistical group. The lowest viability percentage (4.6%) was obtained in the Swan cultivar in the TTC test. The Swan cultivar also exhibited low viability values in the AST test, similar to the TTC test, whereas in the LCB test it showed higher values than the Barolo cultivar. All cultivars exhibited a similar response pattern to the applied staining methods (Fig. 4). Based on the mean values of the viability tests, the highest viability percentage was determined in the SFN test (96.5%, group A), which was statistically superior to all other methods. This was followed by the IKI (90.8%, group B), LCB (82.0%, group C), and AST (76.1%, group D) tests, respectively, while the lowest value was obtained in the TTC test (6.9%, group E). In the IKI test, all cultivars were grouped together, indicating no differences among cultivars. When evaluated on a cultivar basis, the highest viability percentage was determined in the Chic cultivar (71.5%), whereas the lowest was observed in the Swan cultivar (Table 4).

**Table 4:** Comparative pollen viability percentages of chrysanthemum cultivars obtained by different staining assays

Cv.	Viability tests (%)					
	IKI	TTC	AST	SFN	LCB	Avg.
Swan	90.2 ± 1.1 c	4.6 ± 1.1 h	73.4 ± 1.2 f	94.2 ± 0.8 b	83.8 ± 1.1 d	69.2 ± 34.2 c
Barolo	90.1 ± 0.9 c	7.6 ± 1.2 g	78.0 ± 1.1 e	98.0 ± 1.0 a	78.9 ± 1.0 e	70.5 ± 33.5 b
Chic	91.9 ± 1.0 c	8.4 ± 1.4 g	77.1 ± 0.9 e	97.2 ± 1.6 a	83.1 ± 1.1 d	71.5 ± 32.9 a
Avg	90.8 ± 1.1 B	6.9 ± 2.0 E	76.1 ± 2.5 D	96.5 ± 2.1 A	82.0 ± 2.6 C	

*p* ≤ 0.05, the values presented in the table represent non-transformed means ± standard error (SE)

*Cv *Cultivar, *IKI* Iodine potassium iodide, *TTC* 2,3,5-triphenyltetrazolium chloride, *AST* Acetocarmine, *SFN* Safranin, *LCB* Lactophenol cotton blue

**Fig. 5:**
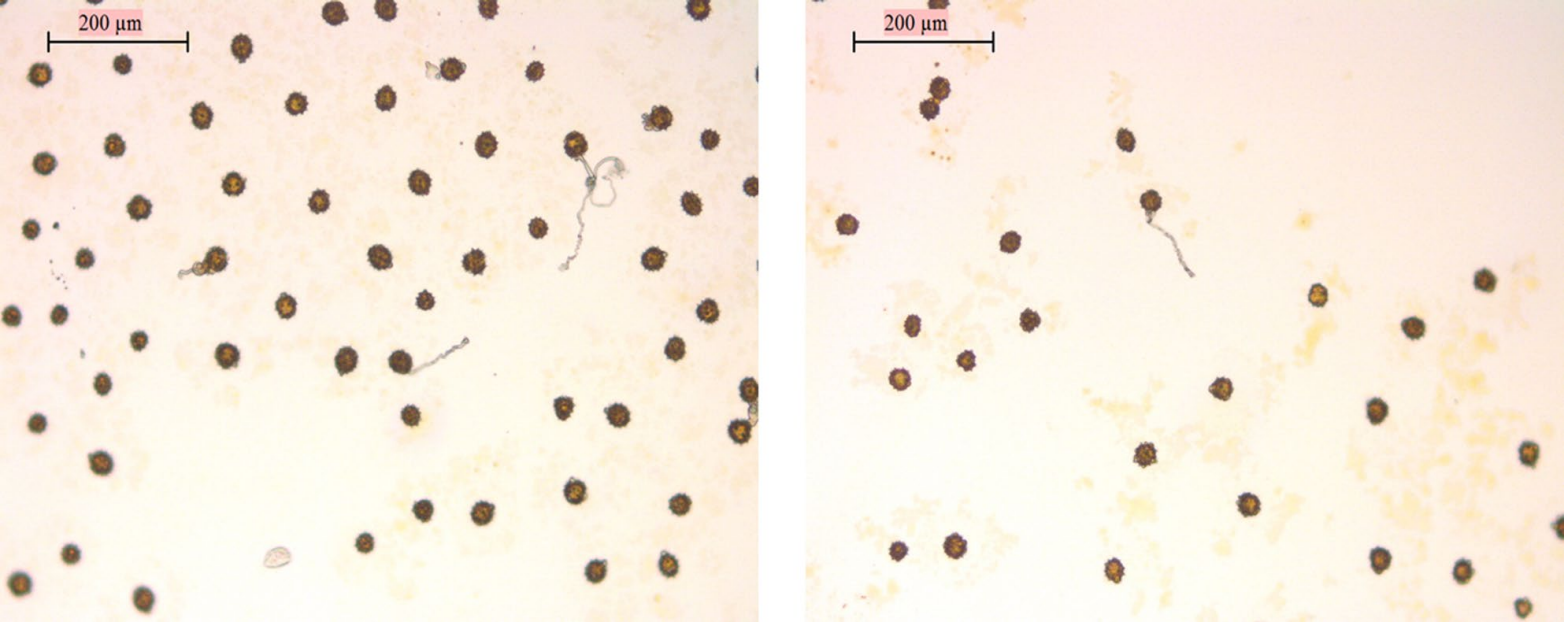
Germinated chrysanthemum pollen grains

Based on the cultivar × germination medium interaction, the highest germination percentage was observed in the S-4 medium in the Swan cultivar (7.3%). In contrast, the lowest value was recorded in the Barolo cultivar in the K-4 medium. Several treatments, including the K-4 and K-3 media, as well as the S-3 and K-1 media for the Swan and Barolo cultivars and S-2 medium for the Chic cultivar, were grouped together, indicating consistently low germination responses under these conditions. Among the germination media, the highest germination percentage was obtained in the S-4 medium (6.5%, group A), which was statistically superior to all other treatments. This was followed by the S-1 medium (4.2%, group B), while the K-2 medium was placed in group C. The S-2, S-3, and K-1 media were grouped in D, whereas the lowest germination percentages were observed in the K-3 and K-4 media (group E), which showed similarly low values. When evaluated by cultivar, the highest germination percentages were recorded in the Swan cultivar, which was grouped together with Chic. In contrast, the Barolo cultivar exhibited the lowest germination percentages. Semi solid media (K group) showed low germination percentages across all cultivars, ranging from 0.1% to 2.8%. In contrast, germination percentages in liquid media (S group) ranged from 0.8% to 7.3%, indicating generally higher germination percentages (Fig. 5; Table 5).

**Table 5:** Comparative pollen germination percentages of chrysanthemum cultivars obtained from different germination tests

Cv	Germination tests (%)									
	S-1	S-2	S-3	S-4	K-1	K-2	K-3	K-4		Avg.
Swan	4.6 ± 1.2c	1.7 ± 0.8dg	0.3 ± 0.1hı	7.3 ± 1.0a	2.4 ± 1.1df	1.3 ± 0.5fı	0.3 ± 0.1hı	0.7 ± 0.2gı		2.3 ± 2.4 A
Barolo	2.5 ± 0.9de	1.1 ± 0.7gı	0.4 ± 0.1hı	6.4 ± 1.2ab	0.4 ± 0.2hı	2.8 ± 1.0d	0.2 ± 0.2hı	0.1 ± 0.1ı		1.7 ± 2.1B
Chic	5.6 ± 1.0bc	0.8 ± 0.2gı	2.9 ± 0.9d	6.0 ± 1.0b	0.7 ± 0.2gı	1.5 ± 0.5eh	0.3 ± 0.2hı	0.2 ± 0.1hı		2.3 ± 2.3 A
Avg	4.2 ± 1.2B	1.2 ± 0.7D	1.2 ± 0.7D	6.5 ± 1.2 A	1.2 ± 0.7D	1.9 ± 1.2 C	0.2 ± 0.2E	0.3 ± 0.2E		

*p* ≤ 0.05, the values presented in the table represent non-transformed means ± standard error (SE)

*Cv *Cultivar, *S-1* Modified liquid ME3-1 medium, *S-2* Modified liquid ME3-2 medium, *S-3* Liquid culture medium, *S-4* PEG-based culture medium, *K-1* Brewbaker and Kwack (BK) medium, *K-2* Modified solid ME_3_ medium, *K-3* Agar-based medium 1, *K-4* Agar-based medium 2

Pollen grains exposed to thermal treatment at 80 °C for 48 h were subjected to viability tests. Among the applied staining methods, TTC did not produce any coloration in the heat-treated pollen, suggesting complete loss of metabolic activity, whereas the other staining methods resulted in partial or even extensive coloration, with staining frequencies exceeding 70%. In addition, when these heat-inactivated pollen grains were transferred onto germination media, none of the tested cultivars exhibited pollen tube emergence in any tested medium (Additional file 1). Since heat treatment resulted in complete loss of pollen germination and TTC staining indicated metabolic inactivation, numerical values were excluded from statistical analysis.

**Fig. 6:**
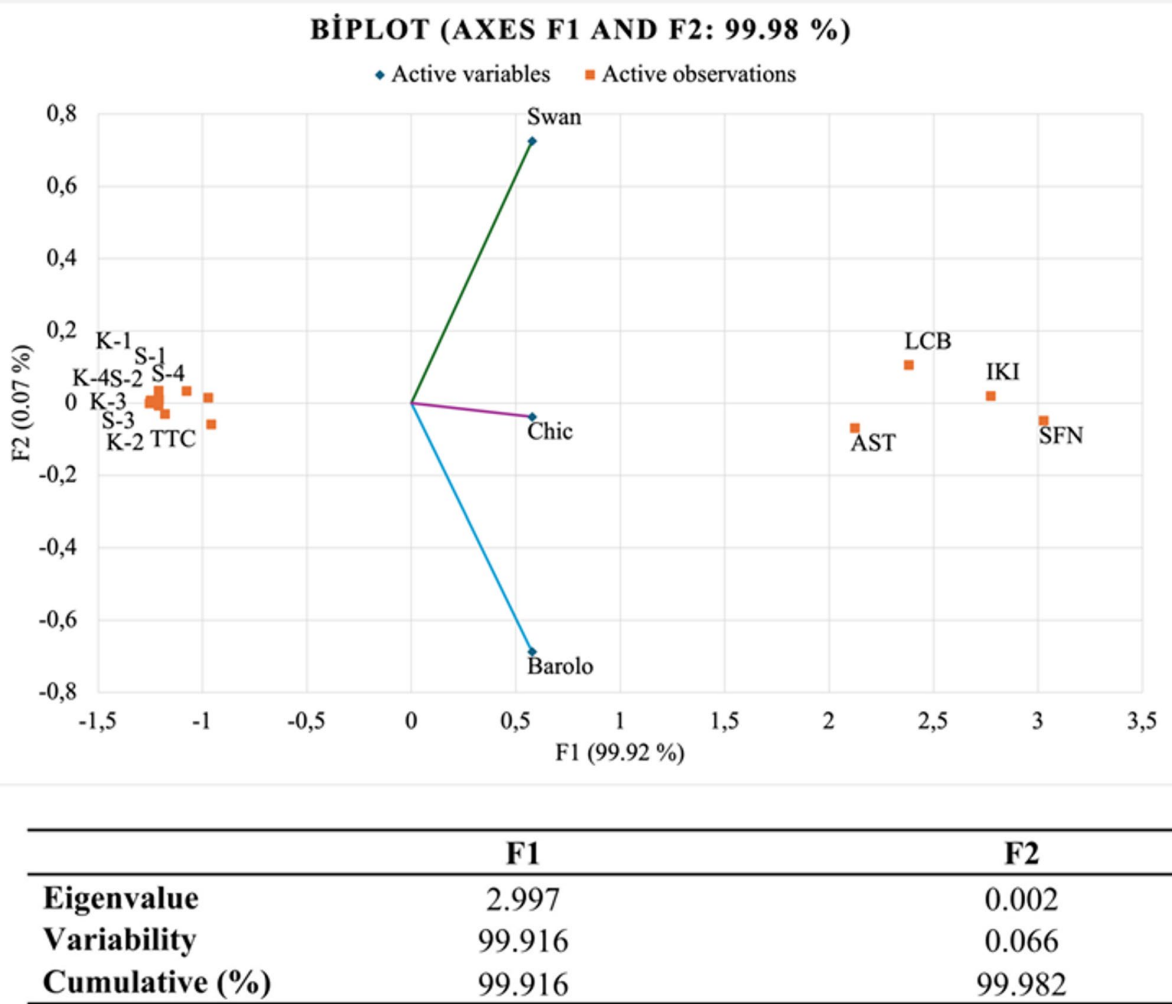
PCA biplot showing the association between pollen viability tests, germination media, and chrysanthemum cultivars. IKI: iodine potassium iodide, TTC: tetrazolium chloride, AST: acetocarmine, SFN: safranin, LCB: lactophenol cotton blue, S-1: modified liquid ME3-1 medium, S-2: modified liquid ME3-2 medium, S-3: liquid culture medium, S-4: PEG-based culture medium, K-1: Brewbaker and Kwack (BK) medium, K-2: modified solid ME_3_ medium, K-3: agar-based medium 1, K-4: agar-based medium 2

Principal Component Analysis (PCA) indicated that 99.9% of the total variance was explained by the first two components. The viability tests SFN, IKI, LCB, and AST clustered together on the positive side of the F1 axis, suggesting a positive association among these tests. In contrast, the TTC test was positioned on the negative side of the F1 axis together with the germination media (S and K groups) and showed a pattern more closely associated with germination data compared to the other viability tests. In terms of cultivars, Swan was located in the positive direction of F2, Barolo in the negative direction, while Chic was positioned near the origin, indicating an intermediate response. Overall, this distribution suggests differences between viability tests and germination behavior, while indicating that TTC may better reflect germination trends compared to the other staining methods (Fig. 6).

## Discussion

The pollen viability and germination results obtained from three *Chrysanthemum × morifolium* cultivars in this study support the previously reported discrepancy between “morphological viability” and “functional viability” in modern chrysanthemums. Although viability percentages in staining tests could reach as high as 98%, germination percentages remained as low as 7.0%. This discrepancy may be explained by the fact that staining tests generally assess structural or morphological integrity and only rarely reflect metabolic activity, whereas pollen germination depends on more complex physiological processes, including ionic balance, membrane permeability, oxidative stability, and energy production [25–27]. Many studies have likewise reported that staining tests often overestimate viability, while functional germination remains low [28–30].

In all cultivars, TTC produced the lowest viability estimates, whereas other staining methods indicated high viability. Despite this, germination percentages remained consistently low across all conditions. This suggests that staining tests such as IKI, AST, LCB, and SFN may overestimate functional germination capacity in chrysanthemum pollen under the conditions tested.

Differences among cultivars were mainly expressed in their responses to the germination media. In certain media, ‘Chic’ achieved the highest percentages, whereas in others ‘Swan’ or ‘Barolo’ produced higher results. This suggests that cultivars may differ in their sensitivity to osmotic conditions, ionic composition, and carbon sources. These differences may be related to variations in physiological characteristics, such as membrane stability, metabolic activity, and pollen structure [31–33]. Accordingly, although staining tests showed similar overall trends across cultivars, false-positive percentages may still vary among them. For example, in cultivars with high starch accumulation, IKI may show increased staining intensity [34]. The relatively higher performance of the ‘Chic’ cultivar in both TTC staining and S-1/S-4 germination media may reflect differences in metabolic activity or physiological stability. In contrast, the lower responses observed in ‘Swan’ and ‘Barolo’ may indicate greater sensitivity to osmotic and ionic conditions.

The variation in germination outcomes among cultivars suggests a genotype × environment interaction, as also supported by the PCA analysis. The positioning of the three cultivars along different PCA axes may indicate that pollen responses to germination media are influenced by genetic factors and may be genotype-dependent. The literature emphasizes that modern chrysanthemum cultivars exhibit wide variation in pollen physiology, likely related to their hexaploid genome structures, high recombination rates, and long selection history [2, 8, 35]. Zhao et al. [36] reported that modern cultivars have much lower pollen fertility than wild species; Miler and Woźny [8] determined a germination percentage of 8.2% in fresh pollen; and Meral et al. [37] reported that germination percentages varied between 3.5% and 16.8% across species and cultivars. Dursun et al. [38] reported germination percentages of 5.2% in the Barolo cultivar and 7.6% in the Chic cultivar, while Kazaz et al. [39] found that germination percentages across 18 cultivars ranged from 11.0% to 22.1%. The germination percentages observed in this study (0.1–7.0%) are generally consistent with these reports, although the lower values observed here are relatively low. This variation may be related not only to genotypic differences but also to methodological factors and growing conditions, which are known to influence pollen viability and germination [40, 41].

Differences among staining tests may be related to the biochemical and histological targets of these methods. IKI stains starch in pollen and can classify pollen as viable even when starch content is preserved but the pollen is metabolically inactivated [42]. SFN is a dye that, due to its positively charged (basic) nature, binds to negatively charged cellular components such as DNA, RNA, and acidic polysaccharides [43–46]; therefore, it may produce intense staining in pollen that preserves cytoplasmic integrity even if it is metabolically inactive. Similarly, AST and LCB target the cytoplasm and thereby evaluate pollen in which membrane integrity is maintained [47]. The common feature of these methods is that they assess pollen viability not as physiological or metabolic capacity, but only through structural integrity. Consequently, these dyes may have limited biological representativeness for determining germination potential. TTC, by contrast, is based on a different biochemical principle and produces formazan primarily in pollen that possesses mitochondrial dehydrogenase activity [47]. For this reason, TTC is sensitive to processes such as energy production, electron transport chain activity, and maintenance of the NADH/NAD⁺ redox balance. Therefore, it may reflect a metabolic threshold that morphological stains may not fully detect [48]. In this study, the markedly lower viability percentages obtained with TTC may result from capturing a biological signal corresponding to the energy-production capacity of chrysanthemum pollen.

Further evidence for this biochemical divergence was observed in pollen killed by heat at 80 °C for 48 h. While heat-inactivated pollen did not stain at all with TTC, it was classified as “viable” at percentages exceeding 70% by stains such as SFN, AST, LCB, and IKI. This finding suggests that TTC may be a more reliable discriminator in pollen grains where structural integrity is maintained but metabolic collapse has occurred. Indeed, the results of this study are consistent with the relatively higher reliability of TTC reported across many species. In *Jatropha curcas* pollen, all methods other than TTC classified dead pollen as viable [18]. In *Leymus chinensis*, IKI stained dead pollen in the same way as fresh pollen [49]. In *Prunus laurocerasus*, TTC was shown to provide a more accurate viability estimate than IKI [50]. This cross-species consistency supports the idea that staining tests in chrysanthemum may not fully reflect functional germination capacity and may reflect a broader physiological pattern observed across species in pollen biology.

Although TTC showed relatively higher agreement with functional viability in this study, Zhao et al. [51] reported that methods such as TTC, modified TTC, IKI, and FDA were not reliable in chrysanthemum pollen. This apparent contradiction may be interpreted as reflecting differences in experimental conditions, particularly considering that chrysanthemum pollen is short-lived [52] and may have low metabolic resilience.

TTC may be particularly sensitive to standardization, including factors such as solution concentration, incubation duration, temperature, and the timing of microscopic evaluation. In particular, formazan formation in TTC is a time-dependent reaction, and optimal results require careful control of time–concentration–temperature parameters; otherwise, both over-staining and under-staining may occur. Therefore, the findings of Zhao et al. [51] may suggest that variability in experimental conditions influences TTC performance, and that under non-optimal conditions, it may yield false-negative results.

This pattern was also supported by PCA, a multivariate analysis. In the PCA, TTC and all germination media were positioned on the same variance axis, whereas IKI, AST, SFN, and LCB clustered together on the opposite axis. This separation suggests that the variance component represented by TTC may be associated with germination behavior, while structural stains may not fully reflect functional viability.

The results obtained from the germination media suggest that chrysanthemum pollen may be highly sensitive to osmotic balance and hydration rate, in addition to metabolic viability. The fact that all liquid media (S-1, S-2, S-3, S-4) produced markedly higher germination percentages than semi-solid media may be associated with the fragile nature of the pollen membrane, which may require controlled and gentle hydration. It has been reported that agar-containing solid and semi-solid media ensure that pollen tubes are distributed properly across the medium and enable better absorption of moisture and microelements; however, as the hardness of the agarized medium increases, it may hinder appropriate exchange of water and micronutrients between pollen and the germination medium and may cause pollen dehydration [53, 54]. In chrysanthemum pollen, agar-containing semi-solid media (in a preliminary experiment, a solid medium was used and no germination was observed) may have limited water and ion diffusion and created mechanical resistance that hindered guided pollen tube growth. In addition, the 0.5% agar level may have been a relatively high concentration for chrysanthemums. Similarly, in rose and lisianthus, liquid media have been reported to yield higher germination percentages than solid and semi-solid media [55, 56]. In our study, the very low germination values produced by the K-series media (0.1–2.8%) suggest that semi solid media are not suitable for in vitro evaluation of chrysanthemum pollen, or that agar should be tested at much lower concentrations.

Although S-4 contained only PEG1500 and a low dose of boric acid, it provided the highest germination percentages for all cultivars. PEG1500 is a high-molecular-weight osmoticum that does not penetrate the pollen membrane; by slowing water entry into the cell, it may help prevent sudden increases in turgor and reduce membrane destabilization [57]. Because chrysanthemum pollen is thought to be structurally prone to rapid hydration stress and disruption of the intine layer due to the combination of a triporate aperture system typical of Asteraceae, a thick exine–thin intine ratio, and highly hydrophilic surface ornamentation, PEG may act as an important buffer in chrysanthemums [58]. It has been reported that PEG regulates plasma membrane permeability in pollen grains and may provide stability to the pollen tube membrane, and the beneficial effects of PEG in germination media have been confirmed across many studies and species [59–61].

The fact that S-4 contains only boric acid is another noteworthy outcome. Boron is known to play an important role in regulating wall elasticity at the pollen tube tip by stabilizing RG-II cross-linking [62], thereby enabling controlled wall expansion during tube growth. Bor also contributes to the regulation of Ca²⁺ channels and to the maintenance of membrane stability. Despite the absence of Ca²⁺ in S-4, the high germination observed may suggest that early-phase Ca²⁺ requirement in chrysanthemum pollen may be met through endogenous stores and the wall–membrane stability provided by boron. Ca²⁺ deficiency may become limiting only at later stages of pollen tube elongation. This is also consistent with findings demonstrating early-phase Ca²⁺ independence in *Malus domestica* pollen [63].

The absence of sucrose in S-4 despite high germination suggests that, in the initial phase, the lack of an external carbon source may not be a primary limiting factor, and that osmotic balance may be a key limiting factor. Similarly, the literature contains evidence showing that the main factor limiting pollen germination is osmotic balance rather than carbon source. In *Petunia hybrida* pollen, a study reported that PEG used as the main component in the germination medium was superior to sucrose: PEG promoted pollen tube growth more than sucrose, and pollen tubes developed on PEG more closely resembled in vivo tubes. Moreover, although pollen germinated on sucrose exhibited a 33% higher respiration rate than pollen germinated on PEG, this increase in energy was not associated with tube elongation; thus, hydration rate and osmotic pressure appear to be more important for tube morphogenesis than sucrose [64]. This may support the view that carbon source alone is not limiting, and that the osmotic structure and phase properties of the medium may be key determinants [64]. In addition, consistent with the classic findings of Brewbaker and Kwack [25] on the roles of Ca²⁺ and boron in pollen tube growth, the low germination observed in the Ca²⁺-containing K-1 medium suggests that the limiting factor may not have been ion composition but rather the phase of the medium. Conversely, low germination in S-3 despite the presence of sucrose suggests that sucrose may have disrupted the osmotic balance of the pollen tube and thereby limited germination. This is consistent with studies reporting that at high concentrations sucrose increases osmotic pressure excessively and disrupts pollen tube polarity and/or that PEG-containing media promote pollen germination more than sucrose [54, 64, 65].

Although S-1 and S-2 contained macro- and microelements together with PEG (200–300 g/L), they did not perform as well as S-4, which may indicate that chrysanthemum pollen may better tolerate “low ion + controlled osmolarity” conditions. Moreover, the PEG4000 used in S-1 may have provided sufficient osmotic buffering to protect chrysanthemum pollen against rapid hydration, whereas the PEG1500 combination in S-2, due to a higher number of molecules and stronger osmotic intensity, may have created an excessively hypertonic environment and likely suppressed pollen tube elongation. Indeed, the osmotic effect of PEG can vary depending on its molecular weight [66]. In other words, a low-molecular-weight PEG1500 medium may have become excessively “dry,” over-restricting water uptake by pollen, whereas a higher-molecular-weight PEG4000 may have provided a more balanced osmotic control without completely preventing water uptake. The superior performance of S-1 relative to S-2 may not be explained solely by differences in total osmotic potential; it may also reflect changes induced by PEG in ion activity and diffusion. Although PEG does not chemically bind ions, changes in water potential and solution properties caused by PEG may reduce the diffusion and availability of mineral ions, thereby influencing their uptake by plant cells. Thus, differences in PEG molecular weight and concentration may influence ion uptake and signaling, which can be important in ion-sensitive systems such as pollen tube growth [67]. Although, at equivalent mass concentrations, higher-molecular-weight PEG solutions (e.g., PEG4000) exhibit higher viscosity than lower-molecular-weight PEG solutions (e.g., PEG1500), very high concentrations of low-molecular-weight PEG can also increase solution viscosity.

From a methodological perspective, it is widely acknowledged that in vitro pollen tests cannot fully represent in vivo conditions: pH microgradients in stylar tissues, ion channels regulating calcium flux, callose barriers, and self-incompatibility mechanisms can direct pollen tube behavior in ways that differ from laboratory conditions. Subjectivity arising from the interpretation of staining intensity and the limited capacity of in vitro systems to model pistil biology represent important methodological limitations of the study. In this context, the findings suggest that a single test is not sufficient for reliable pollen evaluation in chrysanthemum; relying solely on staining tests can lead to errors in male parent selection by overestimating morphological viability relative to functional capacity. The TTC + S-4 combination appeared to provide the most consistent results in this study by jointly assessing metabolic viability and functional germination capacity. The metabolic selectivity of TTC, together with the stable performance of the S-4 medium despite variation among cultivars and germination conditions, suggests that this approach may be considered as a potential evaluation strategy. While the results demonstrate that metabolic viability does not always coincide with functional germination, they also suggest that using TTC-based metabolic tests together with osmotically optimized germination media may contribute to more accurate male parent selection in chrysanthemum breeding programs.

Staining-based viability assessments are observer-dependent, and differences in researchers’ perception and experience may lead to inconsistencies in distinguishing between viable and non-viable pollen grains, thereby introducing potential bias into the results. This issue becomes particularly evident in the evaluation of pollen grains exhibiting borderline staining intensity and may affect the reproducibility of the findings. However, such subjective effects can be minimized through the establishment of standardized evaluation criteria, increased observer training, and the incorporation of more objective analytical methods where possible. Therefore, despite this limitation, staining tests can still provide valuable information for assessing pollen viability when supported by appropriate methodological approaches. In addition, the relatively limited number of cultivars evaluated may restrict the generalizability of the findings to a broader range of chrysanthemum genotypes. The absence of in vivo validation creates uncertainty regarding the extent to which in vitro results reflect pollen performance under natural conditions. Furthermore, the lack of optimization of medium composition and pH conditions may have limited the precise control of environmental factors that directly influence pollen germination. Considering the variable responses of different genotypes to environmental conditions, these factors indicate that the results should be interpreted with caution.

## Conclusion

In conclusion, the present study indicates a discrepancy between staining-based pollen viability estimates and actual germination performance in *Chrysanthemum × morifolium*. Among the evaluated methods, the TTC staining test showed closer agreement with functional pollen viability, while PEG-enriched liquid media applied through the hanging drop technique resulted in higher germination percentages. The combined use of TTC staining and PEG-based germination media may therefore represent a promising approach under the tested conditions. In addition, the results indicate that a simplified germination medium containing only PEG and boric acid may represent a potential alternative to the widely used Monnier medium, although further validation across a wider range of genotypes is required. These findings may provide a useful methodological basis for supporting pollen evaluation and potential male parent assessment in chrysanthemum breeding programs.

## Supplementary Information

Supplementary Material 1.

## Data Availability

All data generated or analysed during this study are included in this published article.

## References

[1] Mekapogu M, Lim SH, Choi YJ, Lee SY, Jung JA. Evaluation of genetic diversity and identification of cultivars in spray-type chrysanthemum based on SSR markers. Genes. 2025;16(1):81. 10.3390/genes1601008. 39858628 10.3390/genes16010081 PMC11764994

[2] Baghele RD. Breeding aspect for improvement in chrysanthemum: A review. Int J Curr Microbiol Appl Sci. 2021;10(5):101–11. 10.20546/ijcmas.2021.1005.015.

[3] Miler N, Kulus D. Effect of parental components and pollination frequency on the setting and germination of chrysanthemum seeds. Horticulturae. 2022;8(9):827. 10.3390/horticulturae8090827.

[4] Teixeira da Silva JA, Kulus D. Chrysanthemum biotechnology: Discoveries from the recent literature. Folia Hortic. 2014;26:67–77. 10.2478/fhort-2014-0007.

[5] Adedeji OS, Naing AH, Kim CK. Protoplast isolation and shoot regeneration from protoplast-derived calli of *Chrysanthemum* cv. White ND. Plant Cell Tissue Organ Cult. 2020;141:571–81.

[6] Haider S, Gao Y, Gao Y. Standardized genetic transformation protocol for *Chrysanthemum* cv. ‘Jinba’ with TERMINAL FLOWER 1 homolog CmTFL1a. Genes. 2020;11:860. 10.3390/genes11080860. 32731555 10.3390/genes11080860 PMC7463584

[7] Datta SK. Induced mutations: Technological advancement for development of new ornamental varieties. Nucleus. 2020;63:119–29.

[8] Miler N, Woźny A. Effect of pollen genotype, temperature and period of storage on in vitro germinability and in vivo seed set in chrysanthemum—Preliminary study. Agronomy. 2021;11(12):2395. 10.3390/agronomy11122395.

[9] Anderson NO, Ascher PD. Inheritance of seed set, germination, and day neutrality/heat delay insensitivity of garden chrysanthemums (*Dendranthema × grandiflora*) under glasshouse and field conditions. J Am Soc Hortic Sci. 2004;129:509–16.

[10] Anderson NO. Chrysanthemum. In: Anderson NO, editor. Flower breeding and genetics. Dordrecht: Springer; 2007. pp. 389–437.

[11] Wang F, Zhang FJ, Chen FD, Fang WM, Teng NJ. Identification of *Chrysanthemum morifolium* self-incompatibility. Sci World J. 2014;2014:625658. 10.1155/2014/625658. 10.1155/2014/625658 PMC3925557 24592176

[12] Yang JS, Endo M. In vitro germination and viability of *Dendranthema* pollen. Asian J Plant Sci. 2005;4:673–7.

[13] Sun CQ, Chen FD, Teng NJ, Liu ZL, Fang WM, Hou XL. Factors affecting seed set in the crosses between *Dendranthema grandiflorum* (Ramat.) Kitamura and its wild species. Euphytica. 2010;171:181–92.

[14] Post A. Ionic composition of the nutrient solution used in chrysanthemum cultivation. Maasdijk, The Netherlands: Deliflor Chrysanten B.V.; 2017.

[15] Kırbay E. Improvement of single cut flower chrysanthemum varieties by cross breeding. PhD dissertation. Ankara University, 2023.

[16] Eti S. A practical method for the determination of pollen production. Çukurova J Agric Food Sci. 1990;5(4):49–58.

[17] Eti S. Determination of pollen viability and germination capability of some fruit species and cultivars by different in vitro tests. Çukurova J Agric Food Sci. 1991;6:69–80.

[18] Abdelgadir HA, Johnson SD, Van Staden J. Pollen viability, pollen germination and pollen tube growth in the biofuel seed crop *Jatropha curcas* (Euphorbiaceae). S Afr J Bot. 2012;79:132–9.

[19] Saarela JM. Taxonomic synopsis of invasive and native *Spartina* (Poaceae, Chloridoideae) in the Pacific Northwest (British Columbia, Washington and Oregon), including the first report of *Spartina × townsendii* for British Columbia. Can PhytoKeys. 2012;10:25–82. 10.3897/phytokeys.10.2734 PMC3310194 22461730

[20] Bolat İ, Pırlak L. An investigation on pollen viability, germination and tube growth in some stone fruits. Turk J Agric For. 1999;23:383–8.

[21] Tingting X. Measurement and marker trait association analysis of pollen viability in the K5 tetraploid rose population. PhD thesis. Wageningen University; 2009.

[22] Dölek-Gencer C, Özkaya MT, Eti S, Karabıyık Ş, Fletcher NTM. Pollen viability and germination levels with the amount of pollen production of some important olive cultivars in Türkiye. Turk J Agric Food Sci Technol. 2023;11(6):1176–82. 10.24925/turjaf.v11i6.1176-1182.5773.

[23] Kumar S, Kumar R, Rajasekharan PE, Dhananjaya MV. Pollen viability and in vitro pollen germination in chrysanthemum (*Dendranthema × grandiflora* Tzvelv). J Hortic Sci. 2024;19(2). 10.24154/jhs.v19i2.3299.

[24] Macovei A, Caser M, Donà M, Valassi A, Giovannini A, Carbonera D, Scariot V, Balestrazzi A. Prolonged cold storage affects pollen viability and germination along with hydrogen peroxide and nitric oxide content in *Rosa hybrida*. Not Bot Horti Agrobo. 2016;44:6–10.

[25] Brewbaker JL, Kwack BH. The essential role of calcium ion in pollen germination and pollen tube growth. Am J Bot. 1963;50:859–65. 10.1002/j.1537-2197.1963.tb06564.x.

[26] Shivanna KR, Sawhney VK. Polyethylene glycol improves the in vitro growth of *Brassica* pollen tubes without loss in germination. J Exp Bot. 1995;46(11):1771–4.

[27] Taylor LP, Hepler PK. Pollen germination and tube growth. Annu Rev Plant Physiol Plant Mol Biol. 1997;48:461–91. 10.1146/annurev.arplant.48.1.461 10.1146/annurev.arplant.48.1.461 15012271

[28] Salomón-Torres R, Elhoumaizi MA, Zambrano-Reyes C, Alboukhari Zaid A, Ruisanchez-Ortega Y, Peña-Yam LP, Gutiérrez-Pacheco MM. Pollen viability and germination assessment after storage in tropical fruit species. Plants. 2025;14(20):3189. 10.3390/plants14203189. 41157746 10.3390/plants14203189 PMC12567252

[29] Soares TL, Silva SO, Souza EH, Jesus ON. Pollen viability and germination of banana diploids and triploids. Rev Bras Frutic. 2013;35(4):1116–25.

[30] Rathod V. Studies on pollen viability and germination in *Momordica* species. J Pharmacogn Phytochem. 2018;6(6):13–7.

[31] Fragallah SA, Xu J, Cai X, Yin Y. Metabolomics analysis reveals clone-specific metabolic changes in Chinese fir (*Cunninghamia lanceolata*) pollen during germination. FTL. 2018;27(4):226–38. 10.1080/14728028.2018.1477813.

[32] Dai H, Chen Y, Liu Z, Zhang Q, Li X. Metabolomics and transcriptomics analysis of pollen germination response to low-temperature storage in pitaya (*Hylocereus polyrhizus*). Front Plant Sci. 2022;13:866588. 10.3389/fpls.2022.866588. 35646022 10.3389/fpls.2022.866588 PMC9134753

[33] Wang J, Kambhampati S, Allen DK, Chen LQ. Comparative metabolic analysis reveals a metabolic switch in mature, hydrated, and germinated pollen in *Arabidopsis thaliana*. Front Plant Sci. 2022;13:836665. 10.3389/fpls.2022.836665. 35665175 10.3389/fpls.2022.836665 PMC9158543

[34] Skrzypkowski W. Pollen development and stainability in *Vicia faba* L. Agriculture. 2023;13(11):2065. 10.3390/agriculture13112065.

[35] Yang W, Glover BJ, Rao G, Yang J. Molecular evidence for multiple polyploidization and lineage recombination in the *Chrysanthemum indicum* polyploid complex (Asteraceae). New Phytol. 2006;171:875–86. 10.1111/j.1469-8137.2006.01779.x. 16918557 10.1111/j.1469-8137.2006.01779.x

[36] Zhao H, Chen F, Fang W, Wang Y, Chen S, Guo W. Study on pollen viability, longevity and pistil receptivity of self-compatible chrysanthemum. Acta Hortic. 2008;766:402.

[37] Meral ED, Kırbay E, Haspolat G, Kazaz S. Evaluation of pollen viability in some spray chrysanthemum varieties during storage period. Ornam Hortic. 2024;30:e242740.

[38] Dursun HB, Kazaz S, Kılıç T. Pollen quality of some spray chrysanthemum varieties at different holding time and temperature. Igdir Univ J Inst Sci Technol. 2023;13(4):2303–14.

[39] Kazaz S, Yanmaz R, Karagüzel Ö, Haspolat G. Improving cut chrysanthemum varieties by cross-breeding and introducing them into the sector. TÜBİTAK Project 119O082. Türkiye; 2023.

[40] Young HJ. Environmental effects on pollen characters and paternity. In: Ottaviano E, Gorla MS, Mulcahy DL, Mulcahy GB, editors. Angiosperm pollen and ovules. New York: Springer; 1984. pp. 445–50. 10.1007/978-1-4612-2958-2_73.

[41] Ramírez-Aliaga P, Foyo-Moreno I, Cariñanos P. Effects of environmental stress on the pollen viability of ornamental tree species in the city of Granada (south-eastern Spain). Forests. 2022;13(12):2131. 10.3390/f1312213.

[42] Melloni MLG, Scarpari MS, Mendonça JR, Perecin D, Landell MGA, Pinto LR. Comparison of two staining methods for pollen viability studies in sugarcane. Sugar Tech. 2013;15(1):103–7. 10.1007/s12355-012-0185-6.

[43] Anonymous. Safranin staining. New Dev Chem. 2025. Available from: https://openaccesspub.org/new-developments-in-chemistry/safranin-staining

[44] Kuhlmann WD. Dyes, stains, and special probes in histology. Laboratory Diagnostics & Cell Science, D-56112 Lahnstein. 2024. Available from: https://www.kuhlmann-biomed.de/wp-content/uploads/2023/06/IET_introduct_05.pdf

[45] Preethi S, Sivasamy A, Sivanesan S, Ramamurthi V, Swaminathan G. Removal of safranin basic dye from aqueous solutions by adsorption onto corncob activated carbon. Ind Eng Chem Res. 2006;45(22):7627–32. 10.1021/ie0604122.

[46] Lipfert J, Doniach S, Das R, Herschlag D. Understanding nucleic acid–ion interactions. J Am Chem Soc. 2014;136(10):3839–47. 10.1146/annurev-biochem-060409-092720 PMC4384882 24606136

[47] Stokes M, Geitmann A. Screening methods for thermotolerance in pollen. Ann Bot. 2025;135(1–2):71–88. 10.1093/aob/mcae067. 38712364 10.1093/aob/mcae067 PMC11979752

[48] Rich PR, Mischis LA, Purton S, Wiskich JT. The sites of interaction of triphenyltetrazolium chloride with mitochondrial respiratory chains. FEMS Microbiol Lett. 2001;202(2):181–7. 10.1111/j.1574-6968.2001.tb10801.x. 11520612 10.1111/j.1574-6968.2001.tb10801.x

[49] Huang Z, Zhu J, Mu X, Lin J. Pollen dispersion, pollen viability and pistil receptivity in *Leymus chinensis*. Ann Bot. 2004;93(3):295–301. 10.1093/aob/mch044. 14744707 10.1093/aob/mch044 PMC4242205

[50] Sulusoglu M, Cavusoglu A. In vitro pollen viability and pollen germination in cherry laurel (*Prunus laurocerasus* L). Sci World J. 2014;2014:657123. 10.1155/2014/657123. 10.1155/2014/657123 PMC4227381 25405230

[51] Zhao H, Chen F, Fang W. Detection methods of pollen viability of *Dendranthema*. J Zhejiang A&F Univ. 2006;23(4):406–9.

[52] Zhao H, Chen F, Fang W. Pollen germination in vitro of *Chrysanthemum* cultivars with small inflorescences and several species of *Dendranthema*. J Nanjing Agric Univ. 2005;28:22–7.

[53] Connor KF, Towill LE. Pollen-handling protocol and hydration/dehydration characteristics of pollen for application to long-term storage. Euphytica. 1993;68:77–84.

[54] Brandoli C, Cristofori V, Silvestri C, Todeschini C, Sgarbi E. The development of an improved medium for the in vitro germination of *Corylus avellana* L. pollen. Forests. 2024;15(7):1095. 10.3390/f15071095.

[55] Wrońska-Pilarek D, Tomlik-Wyremblewska A. Pollen viability and in vitro germination of selected Central European species from genus *Rosa* analysed with different methods. Dendrobiology. 2010;64:43–53.

[56] Kılıç T, Sinanoğlu E, Kırbay E, Kazaz S, Ercişli S. Determining appropriate methods for estimating pollen viability and germination rates in lisianthus. Acta Sci Pol Hortorum Cultus. 2024;23(3):33–42. 10.24326/asphc.2024.5378.

[57] Sajid ZA, Aftab F. Improvement of polyethylene glycol-, sorbitol-, mannitol-, and sucrose-induced osmotic stress tolerance through modulation of polyamines, proteins, and superoxide dismutase activity in potato. Int J Agron. 2022;2022:5158768. 10.1155/2022/5158768.

[58] Patil SA, Nimbalkar MS, Pagariya MC, Kulkarni AJ, Jadhav PR, Mane MP, Magdum AB, Saha TN, Shinde KV, Prasad KV, Dixit GB, Kawar PG. Pollen morphology and variability among Indian cultivars of *Chrysanthemum morifolium* and comparative analysis with genera of the Asteraceae family. Genet Resour Crop Evol. 2025;72(2):2227–47. 10.1007/s10722-024-02094-0.

[59] Ćalić D, Devrnja N, Kostić I, Kostić M. Pollen morphology, viability, and germination of *Prunus domestica* cv. Požegača Sci Hortic. 2013;155:118–22. 10.1016/j.scienta.2013.03.017.

[60] Jayaprakash P. Pollen germination in vitro. In: IntechOpen. 2018. 10.5772/intechopen.75360

[61] Weng Z, Deng Y, Tang F, Zhao L, Wang Y, Dai X, Zhou Z, Cao Q. Screening and optimisation of in vitro pollen germination medium for sweetpotato (*Ipomoea batatas*). Plant Methods. 2023;19:93. 10.1186/s13007-023-01050-w. 37644497 10.1186/s13007-023-01050-w PMC10463589

[62] Dumont M, Lehner A, Bouton S, Kiefer-Meyer MC, Voxeur A, Pelloux J, Lerouge P, Mollet JC. The cell wall pectic polymer rhamnogalacturonan-II is required for proper pollen tube elongation. Ann Bot. 2014;114(6):1177–88. 10.1093/aob/mcu093. 24825296 10.1093/aob/mcu093 PMC4195553

[63] Fang K, Gao S, Zhang W, Xing Y, Cao Q, Qin L. Addition of phenylboronic acid to *Malus domestica* pollen tubes alters calcium dynamics, disrupts actin filaments and affects cell wall architecture. PLoS ONE. 2016;11(2):e0149232. 26886907 10.1371/journal.pone.0149232 PMC4757038

[64] Zhang HQ, Croes AF. A new medium for pollen germination in vitro. Acta Bot Neerl. 1982;31(1–2):113–9. 10.1111/j.1438-8677.1982.tb01597.x.

[65] Loguercio LL. Pollen treatment in high osmotic potential: A simple tool for in vitro preservation and manipulation of viability in gametophytic populations. Braz J Plant Physiol. 2002;14(1):33–8. 10.1590/S1677-04202002000100009.

[66] Steuter AA, Mozafar A, Goodin JR. Water potential of aqueous polyethylene glycol. Plant Physiol. 1981;67(1):64–7. 10.1104/pp.67.1.64. 16661635 10.1104/pp.67.1.64 PMC425622

[67] Castañeda-Castro O, Gómez-Merino FC, Trejo-Téllez LI, Pastelín-Solano MC. Osmotic stress induced by polyethylene glycol alters macronutrient concentrations in sugarcane (*Saccharum* spp.) plants in vitro. Agrociencia. 2015;49:859–73.

