# Supplementary material for: Reliable assessment of pollen quality in Chrysanthemum × morifolium: comparative evaluation of viability tests and germination media
Source: BMC Plant Biol. 2026 Jun 8;26:1373. doi: 10.1186/s12870-026-09032-5 (PMC13471577; doi:10.1186/s12870-026-09032-5)
Supplement: Supplementary file 1 [file 12870_2026_9032_MOESM1_ESM.pdf]

Additional Table 1. Viability and germination percentages of heat-treated pollen in chrysanthemum cultivars

| Cv. | Viability tests (%) | | | | | |
|---|---|---|---|---|---|---|
| | IKI | TTC | AST | SFN | LCB | Avg. |
| **Swan** | 65.0 | 0 | 68.0 | 74.0 | 70.0 | 55.4 |
| **Barolo** | 71.0 | 0 | 54.0 | 58.0 | 63.0 | 49.2 |
| **Chic** | 52.0 | 0 | 56.0 | 62.0 | 68.0 | 47.6 |
| **Avg** | 62.7 | 0 | 59.3 | 64.7 | 67.0 | |

| Cv | Germination tests (%) | | | | | | | |
|---|---|---|---|---|---|---|---|---|
| | S-1 | S-2 | S-3 | S-4 | K-1 | K-2 | K-3 | K-4 |
| **Swan** | 0 | 0 | 0 | 0 | 0 | 0 | 0 | 0 |
| **Barolo** | 0 | 0 | 0 | 0 | 0 | 0 | 0 | 0 |
| **Chic** | 0 | 0 | 0 | 0 | 0 | 0 | 0 | 0 |

Cv: cultivar, IKI: iodine potassium iodide, TTC: 2,3,5-triphenyltetrazolium chloride, AST: acetocarmine, SFN: safranin, LCB: lactophenol cotton blue. S-1: Modified liquid ME3-1 medium, S-2: Modified liquid ME3-2 medium, S-3: Liquid culture medium, S-4: PEG-based culture medium, K-1: Brewbaker and Kwack (BK) medium, K-2: Modified solid $ME_3$ medium, K-3: Agar-based medium 1, K-4: Agar-based medium 2.
